# Genetic studies in mice directly link oocytes produced during adulthood to ovarian function and natural fertility

**DOI:** 10.1038/s41598-017-10033-6

**Published:** 2017-08-30

**Authors:** Ning Wang, Chonthicha Satirapod, Yasuyo Ohguchi, Eun-Sil Park, Dori C. Woods, Jonathan L. Tilly

**Affiliations:** 1 Vincent Center for Reproductive Biology, Department of Obstetrics and Gynecology, Massachusetts General Hospital, Boston, 02114 Massachusetts USA; 2 Department of Obstetrics, Gynecology and Reproductive Biology, Harvard Medical School, Boston, Massachusetts, 02115 USA; 3 Department of Biology, Laboratory of Aging and Infertility Research, Northeastern University, Boston, Massachusetts, 02115 USA; 4 Genetics Division, Department of Medicine, Brigham and Women’s Hospital, Harvard Medical School, Boston, Massachusetts, 02115 USA

## Abstract

Multiple labs have reported that mammalian ovaries contain oogonial stem cells (OSCs), which can differentiate into oocytes that fertilize to produce offspring. However, the physiological relevance of these observations to adult ovarian function is unknown. Here we performed targeted and reversible ablation of premeiotic germ cells undergoing differentiation into oocytes in transgenic mice expressing the suicide gene, *herpes simplex virus thymidine kinase* (*HSVtk*), driven by the promoter of *stimulated by retinoic acid gene 8* (*Stra8*), a germ cell-specific gene activated during meiotic commitment. Over a 21-day ablation phase induced by the HSVtk pro-drug, ganciclovir (GCV), oocyte numbers declined due to a disruption of new oocyte input. However, germ cell differentiation resumed after ceasing the ablation protocol, enabling complete regeneration of the oocyte pool. We next employed inducible lineage tracing to fate map, through Cre recombinase-mediated fluorescent reporter gene activation only in *Stra8*-expressing cells, newly-formed oocytes. Induction of the system during adulthood yielded a mosaic pool of unmarked (pre-existing) and marked (newly-formed) oocytes. Marked oocytes matured and fertilized to produce offspring, which grew normally to adulthood and transmitted the reporter to second-generation offspring. These findings establish that oocytes generated during adulthood contribute directly to ovarian function and natural fertility in mammals.

## Introduction

Male germline stem cells (GSCs), or spermatogonial stem cells (SSCs), have been identified in the testes of essentially all animal species^1, 2^. The existence of female GSCs, or oogonial stem cells (OSCs), in adult ovaries has been established and is now widely accepted for flies^3^ and fish^4^. Until recently, however, it was thought that female mammals relied on primordial germ cells to generate their entire quota of oocytes during embryogenesis. As such, female GSC function in higher vertebrates was thought to be lost during fetal development, leading to the endowment of a non-renewable pool of ‘resting’ or quiescent primordial oocyte-containing follicles at birth^5^. Once established, the continuous exit of primordial follicles from this resting pool, due primarily to growth activation to primary follicle stages and beyond – a process in mice that has been estimated to ‘deplete’ the primordial oocyte stockpile by approximately 89 follicles per day during postnatal life^6^, eventually leads to complete follicular exhaustion as females reach advanced reproductive ages^7, 8^. This paradigm of a non-renewing pool of oocytes was questioned by a study in 2004 that identified mitotically-active cells expressing DEAD box polypeptide 4 (Ddx4), a conserved germ cell marker^9^, in ovaries of juvenile and young adult mice^10^. Additionally, histomorphometry-based counting of viable and atretic oocytes over time, coupled with mathematical modeling, uncovered a pronounced discordance in how quickly primordial oocyte numbers should decline during postnatal life, if this pool is non-renewing, versus what occurs, which is much slower^10^. This discordance, verified by others later^11^, raised questions over how the primordial oocyte population, if fixed at birth^5^, can remain relatively unchanged in numbers during juvenile and young adult life in the face of a constant rate of exit (depletion) through growth activation^6^.

In 2009, OSCs were isolated from postnatal mouse ovaries using Ddx4 antibody-based sorting^12^. In addition to reaffirming the mitotic capacity and other characteristic features of these primitive germ cells reported earlier^10^, this study also showed that GFP-expressing OSCs transplanted into ovaries of chemotherapy-conditioned wild type mice undergo differentiation into oocytes that mature into eggs, which can be fertilized to produce viable offspring^12^. Many reports followed describing the existence and characteristics of OSCs in ovaries of not just mice, but also rats, cows, non-human primates and humans^13–35^. Paradigms shifts are not without controversy, however, and the identification of OSCs in mammals is no exception. One area of debate concerns the strategy used to obtain OSCs for characterization studies. The Wu lab first published on the utility of Ddx4 antibody-based magnetic-assisted cell sorting (MACS) to isolate OSCs from mouse ovaries^12^. These observations were subsequently confirmed and extended by others with the validation of Ddx4 antibody-based fluorescence-activated cell sorting (FACS) for OSC isolation^16–20, 22, 26–32^. Nonetheless, conceptual issues have been raised by some scientists disputing the existence of OSCs in mammals based on claims that antigenic sequences in Ddx4 should not be useful for antibody-based sorting of viable OSCs if Ddx4 is a cytoplasmic protein in germ cells^36–38^, as reported previously^9, 39^. However, these previous reports were published a decade or more prior to the initial purification of OSCs, and thus conclusions drawn regarding Ddx4 localization in germ cells were derived from analysis of embryonic (primordial) germ cells, male germ cells or oocytes, not OSCs.

Following the first report of OSC isolation^12^, antibody-conjugated microbead technology confirmed that Ddx4 is retained completely inside of oocytes; however, parallel analysis of OSCs identified externalization of the C-terminus of Ddx4 protein^16^. Extensive FACS-based validation work further showed that extracellular Ddx4 (ecDdx4)-positive cells isolated from adult mouse ovaries through C-terminal antibody binding to viable (non-permeabilized) cell fractions are, in turn, recognized by a different (N-terminal) Ddx4 antibody only after the purified cells are permeabilized^16^. These types of dual antibody-single protein studies, which are standard practice for identification of cell surface antigens^40^, not only established the specificity of both antibodies used for analysis of Ddx4 but also the extracellular (C-terminus) versus intracellular (N-terminus) location of different antigenic sequences of Ddx4 in OSCs. Moreover, since both Ddx4 antibodies specifically labeled oocytes in fixed ovarian tissue sections^16^, the reported inability of C-terminal Ddx4 antibodies to recognize viable oocytes during FACS is due to an absence of this antigenic sequence on the surface of non-permeabilized oocytes – a conclusion supported by antibody-conjugated microbead studies^16^. It is worth noting that a year before the first report of OSC isolation by Ddx4 antibody-based sorting^12^, viable germ cells were purified from cultures of human embryonic stem cells using FACS coupled with DDX4 antibodies^41^.

The existence of OSCs in mammalian ovaries has been documented by other means of purification as well. For example, *OG2* transgenic mice [also referred to as *Tg*(*Pou5f1-EGFP*)*2Mn* or Δ*PE-Oct4-Gfp* transgenic mice], with expression of enhanced green fluorescent protein (EGFP) driven by a modified *POU domain class 5 transcription factor 1* (*Pou5f1*; also referred to as *octamer-binding transcription factor-4* or *Oct-4*) gene promoter fragment to convey germline specificity, have been used to obtain OSCs from postnatal ovarian tissue^13^. The cells display the hallmark features of OSCs isolated by Ddx4 antibody-based sorting, including a germline gene expression profile, mitotic capacity and growth *in vitro*, and the ability of OSC-derived oocytes to interact with endogenous ovarian granulosa cells to form follicles^13^. Another strategy involves immunological sorting with antibodies directed against an undisputed transmembrane-spanning protein in germ cells, interferon-induced transmembrane protein 3 (Ifitm3; also referred to as Fragilis)^42, 43^. Expression of *Ifitm3* is used frequently as an endpoint in studies of primitive germ cells, and Ifitm3 antibody-based sorting of embryonic primordial germ cells is well documented^44, 45^. Expression of *Ifitm3* at both the mRNA and protein levels can be detected in OSCs of multiple species^12, 16, 21, 30^, and antibodies against an extracellular domain of Ifitm3 have been used to sort OSCs from mouse^14, 46^ and rat^21^ ovaries. These cells, like those sorted using Ddx4 antibodies, generate functional eggs and offspring following transplantation^21^.

Despite these many advances in the study of OSCs and postnatal oocyte formation over the past decade or so, the physiological significance of *de-novo* oogenesis in the ovaries of adult female mammals remains unknown. One approach for determining the *in-vivo* function of a specific cell type or process is suicide gene technology. Suicide genes, such as *herpes simplex virus thymidine kinase* (*HSVtk*), encode enzymes that are inert in mammalian cells. However, in the presence of target pro-drugs, these enzymes generate cytotoxic metabolites that kill the suicide gene-expressing cells. As examples, suicide gene transgenic mice have been used to define the function of pituitary somatotropes^47^, neural progenitors^48^ and bone marrow osteoblasts^49^ following selective ablation *in vivo*. A second widely-accepted approach for determination of cell function *in vivo* is genetic lineage tracing, which uses a cell type-specific promoter to permanently ‘mark’, at both the genomic (recombination) and phenotypic (reporter gene expression) levels, a desired cell in the body and then map its fate. Past studies of hematopoiesis^50^, neurogenesis^51^, intestinal crypt cells^52^, muscle^53^, hair follicles^54^, and female GSCs in the teleost medaka^4^ provide examples of the use of this technology. Herein we sought to combine these two powerful genetic approaches to rigorously explore the contribution, if any, of postnatal oogenesis to adult ovarian function and female fertility in mammals.

## Results

### Transplanted OSCs generate offspring

Intragonadal transplantation of SSCs expressing a marker gene that can be traced through spermatogenesis to progeny by genotype analysis, a technique first developed over 20 years ago^55, 56^, remains to this day the undisputed gold standard for establishment of male GSC identity and function^57^. In 2009, the generation of offspring derived from GFP-expressing OSCs transplanted into the ovaries of wild type female mice was reported^12^. This outcome, which achieved the exact same bar for functional identity testing of SSCs used without debate for decades^55–57^, has not only been confirmed in mice and extended to rats by this same group^15, 21, 34, 35^, but has also been verified by others^25^. As a preface to embarking on studies of the physiological relevance, if any, of OSCs and oogenesis to adult female reproductive function, we independently assessed this experimental paradigm once again. We used young adult *OG2* transgenic female mice, which are well characterized and widely utilized in studies of germ cell development due to the restricted expression of EGFP in the germline^58–61^, for OSC isolation and intraovarian transplantation into ovaries of young adult wild type recipients^16^. Past studies have already demonstrated that *OG2* transgenic OSCs differentiate into EGFP-positive oocytes that interact with granulosa cells to form follicles both *in vitro* ^13^ and *in vivo* ^62^. In natural mating trials, 4 transplanted wild type females mated with wild type males delivered a total of 38 offspring over the duration of our study period, 6 of which (15.8%) carried the *OG2* transgene and thus were derived from the transplanted OSCs (Supplementary Fig. S1). Of the 4 transplanted females, 3 delivered at least one transgenic pup over the course of the mating trial. Although repeated confirmation of the reproducibility of this outcome is important, intragondal GSC transplantation-based approaches – whether conducted in males^55–57^ or females^15, 21, 25, 34, 35^ (Supplementary Fig. S1), all suffer from the same major interpretational limitation: the data obtained do not provide insight into the potential contribution of GSCs to adult gonadal function and fertility under normal physiological conditions.

### Targeted ablation of differentiating germ cells: validation and controls

Since meiosis is a cellular differentiation process unique to the germline, we next designed a suicide gene-based targeting strategy in mice using a well-characterized 1.4-kb fragment of the promoter of *stimulated by retinoic acid gene 8* (*Stra8*), a germ cell-specific gene activated during meiotic entry in both male and female mice^63–67^. Our selection of this specific region of the *Stra8* promoter offers not only germ cell expression specificity in transgenic animals^18, 68^, but also the advantage of a brief and defined window of activation during the early meiotic commitment phase of GSC differentiation^18, 63–68^. We considered targeting OSCs directly; however, the lack of a candidate gene with restricted expression in OSCs and not other stem cells or more differentiated germ cells precluded this. This strategy would also not permit phenotype-reversibility studies following suicide gene pro-drug exposure and removal since the originating stem cells would be ablated, and thus unavailable to potentially restore the oocyte-generating (oogenic) pipeline once pro-drug treatment was ceased. Although there are several genes that show restricted expression in oocytes^69^, targeted ablation of these terminal cells in the female germ cell differentiation program would obscure data interpretation when changes in oocyte numbers represent the readout for *de-novo* oogenesis. Use of this well-characterized *Stra8* promoter fragment to restrict, in *pStra8-HSVtk* transgenic mice, the cytotoxic actions of *HSVtk* pro-drug exposure to only early differentiating germ cells formed from OSCs prior to oocyte generation, without targeting OSCs or oocytes directly, would circumvent these technical and interpretational limitations. This would therefore enable us to clearly assess the *in-vivo* significance, if any, of active oogenesis to adult ovarian function.

**Figure 1:**
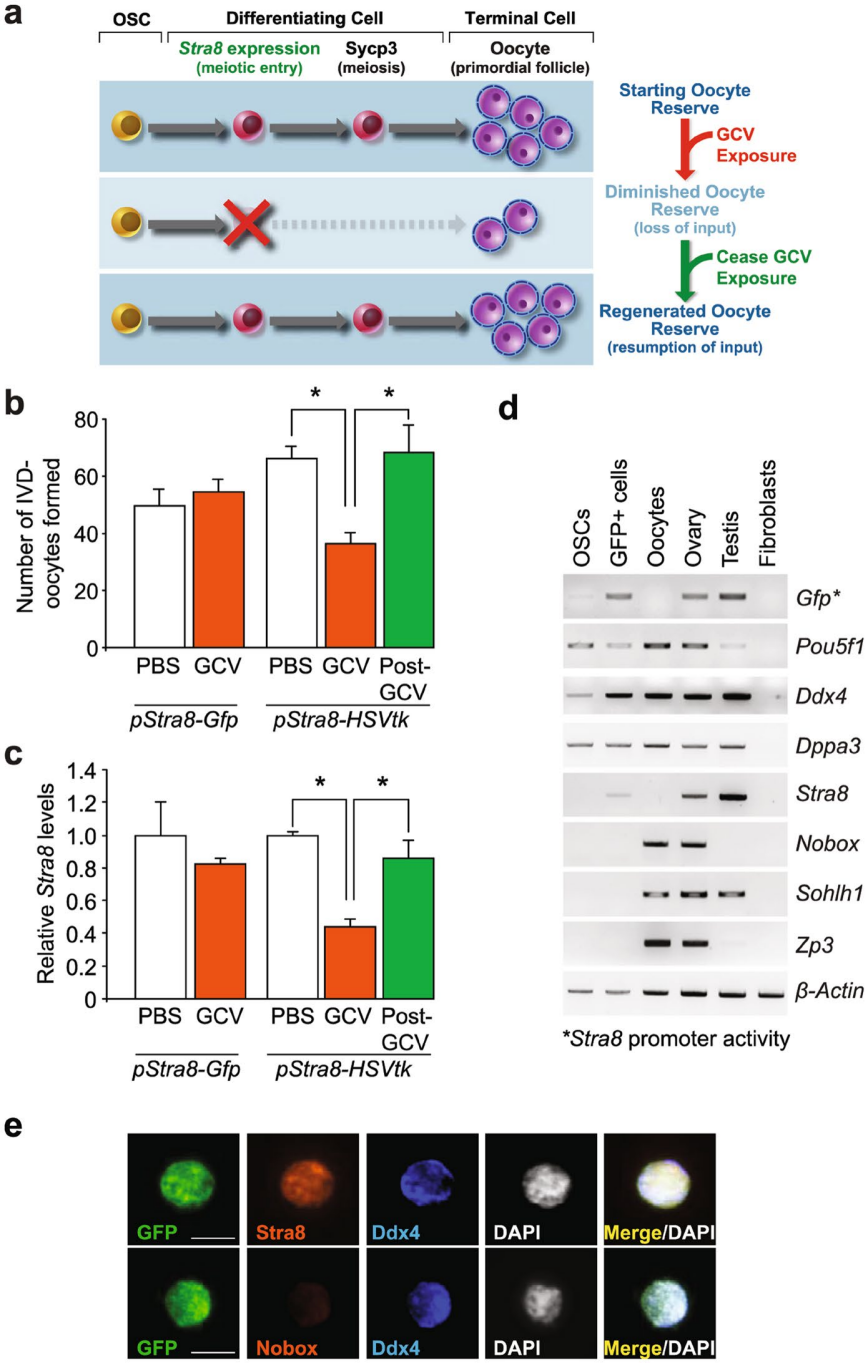
Temporal and targeted disruption of OSC differentiation leads to reversible oogenic failure *in vitro*. (**a**) Schematic depiction of the inducible *HSVtk* suicide gene approach for targeted ablation of female germ cells committing to meiosis, and the expected outcome of GCV exposure and removal on oogenesis. (**b**) Number of IVD-oocytes formed by OSCs expressing *pStra8-Gfp* or *pStra8-HSVtk* 48 h after passage and seeding 2.5 × 10^4^ cells per well in 24-well culture plates in the absence (PBS, vehicle) or presence of GCV (2 μM); the post-GCV group depicts *pStra8-HSVtk*–expressing OSCs cultured with GCV, washed and re-seeded as described above in PBS for assessment of oogenesis 48 h later (mean ± s.e.m., *n* = 3 independent cultures; *P < 0.05). (**c**) Changes in endogenous *Stra8* expression in OSC cultures described in panel b (mean ± s.e.m., *n* = 3 independent cultures; *P < 0.05). (**d**) Representative analysis of OSCs, GFP-expressing ovarian cells, oocytes, ovaries, testes, or adult tail-snip fibroblasts isolated from adult *pStra8-Gfp* transgenic mice for expression of *Stra8*-promoter driven expression of *Gfp*, germ cell markers (*Pou5f1*, *Ddx4*, *Dppa3*), endogenous *Stra8*, oocyte markers (*Nobox*, *Sohlh1*, *Zp3*; *Sohlh1* is also known to be expressed in male germ cells), or *β-actin*. Complete (uncropped) PCR gels for each target sequence amplified are shown in Supplementary Fig. S3. (**e**) Representative immunofluorescence analysis of individual GFP-positive cells purified by FACS from ovaries of *pStra8-Gfp* mice for expression of GFP, Stra8, Nobox or Ddx4 proteins (DAPI nuclear stain, *white*; scale bars, 10-μm).

Two *Stra8* promoter-driven transgene constructs were prepared: one to drive expression of *HSVtk* (*pStra8-HSVtk*) (Supplementary Fig. S2), and another to drive expression of GFP (*pStra8-Gfp*) for use as a control^18^. To initially test if reversible disruption of differentiation impairs the ability of OSCs to generate oocytes (Fig. 1a), OSCs were purified from ovaries of 2-month-old female mice and established in culture^16, 17^. Once OSC lines with stable expression of *pStra8-Gfp* or *pStra8-HSVtk* were obtained following transfection and G418 selection, the cells were treated with vehicle or the HSVtk pro-drug, ganciclovir (GCV), for 4 days and then split at low density to assess spontaneous formation of *in vitro*-derived (IVD)-oocytes^16, 17, 19^. Because IVD-oocytes are poduced in pure germ cell cultures lacking any type of naturally occurring somatic cells (*viz*., granulosa cells) that are crucial for orchestrating key stages of meiotic arrest required for endogenous oocytes to successfully complete normal maturation, IVD-oocytes are not functional in the sense of fertilization or developmental competency. Nonetheless, this *in-vitro* culture approach provides a quick, inexpensive and reliable bioassay to study OSC differentiation under experimentally defined conditions^16, 17, 19, 33, 35^. In vehicle-treated *pStra8-HSVtk* OSC cultures, the rate of IVD-oocyte formation was comparable to that observed in *pStra8-Gfp* OSCs cultured with vehicle (Fig. 1b). In cultures of *pStra8-Gfp* OSCs, used as a negative control to rule out non-specific actions of GCV, IVD-oocyte formation was unaffected by GCV treatment; however, parallel treatment of *pStra8-HSVtk* OSCs with GCV caused a significant attenuation of IVD-oocyte formation (Fig. 1b). This effect was reversible in that the number of IVD-oocytes produced in *pStra8-HSVtk* OSC cultures returned to control levels after GCV was removed (Fig. 1b). Endogenous *Stra8* expression paralleled the pattern of IVD-oocyte formation in response to GCV exposure and removal (Fig. 1c). These results provided a strong impetus for us to then generate the *pStra8-HSVtk* transgenic mouse line for evaluation of adult oogenesis *in vivo*.

To eliminate potential confounding effects of random transgene integration and variability in copy number associated with pronuclear injection, we introduced our transgenes into the neutral *Hprt* genomic locus for generation of the mouse lines^18^. In mice, transgene expression controlled by this 1.4-kb fragment of the *Stra8* promoter is restricted to the gonads, and more specifically to germ cells undergoing meiotic differentiation^18, 68^ (Supplementary Fig. S2). Although the *Stra8* gene is not actively expressed in mouse oocytes^64–67, 70^, we felt it was still important for clear data interpretation in subsequent experiments to characterize the *Stra8* promoter-positive cell fraction in ovaries of adult *pStra8-Gfp* mice to verify this. Gene expression analysis showed that both ecDdx4-positive cells (*i.e*., OSCs) and GFP-positive cells purified independently by FACS from dispersed ovaries of adult *pStra8-Gfp* mice expressed the pluripotent stem cell marker *Pou5f1*, as well as the germ cell markers *Ddx4* and *developmental pluripotency-associated 3* (*Dppa3*) (Fig. 1d and Supplementary Fig. S3). The *Stra8* promoter-driven GFP-positive ovarian cells also contained endogenous *Stra8* mRNA as well as *Gfp* mRNA – the latter indicative of *Stra8* promoter activation, whereas no expression of either endogenous *Stra8* or the *Stra8* promoter-driven *Gfp* transgene was detected in freshly purified OSCs or, importantly, in isolated oocytes (Fig. 1d and Supplementary Fig. S3). These findings confirmed that the 1.4-kb *Stra8* promoter fragment used does not direct transgene expression in oocytes, consistent with prior reports that *Stra8* is shut off once oocytes are formed^64–67, 70^.

In further support of this, *pStra8-Gfp*–positive ovarian cells did not express any markers specific for primordial or early growing immature oocytes (*newborn ovary homeobox* or *Nobox*; *spermatogenesis and oogenesis helix-loop-helix factor 1* or *Sohlh1*; *zona pellucida glycoprotein 3* or *Zp3*)^69, 71–73^, whereas expression of all three of these genes was readily detected in isolated oocytes and in whole ovaries containing oocytes (Fig. 1d and Supplementary Fig. S3). As an additional confirmation that the 1.4-kb *Stra8* promoter fragment used for transgenic mouse generation is neither active in oocytes nor drives transgene expression in oocytes *in vivo*, single-cell immunofluorescence analysis of GFP-expressing cells freshly sorted from ovaries of adult *pStra8-Gfp* mice demonstrated the presence of Ddx4 and endogenous Stra8 proteins but an absence of the well-characterized primordial oocyte protein, Nobox (Fig. 1e). These experiments, taken collectively, documented the fidelity of targeting female germ cells (*Pou5f1-*, *Dppa3-* and *Ddx4*-positive) that are committing to meiosis (*Stra8*-positive; promoter activity and endogenous gene), but have not yet completed differentiation into newly formed oocytes (*Nobox-*, *Sohlh1-* and *Zp3*-negative), by using this specific *Stra8* promoter fragment as a driver for *in-vivo* transgene expression in mice.

**Figure 2:**
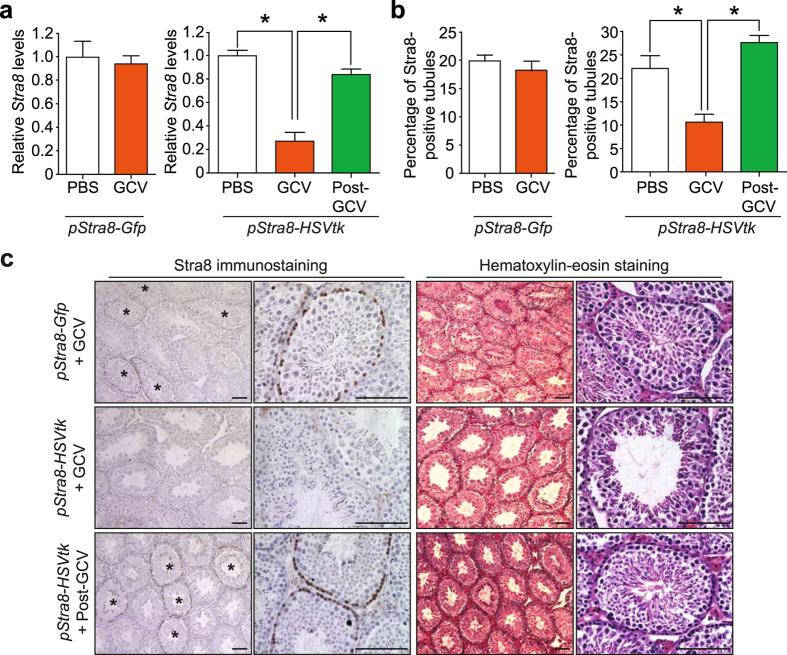
Reversible spermatogenic failure in adult male *pStra8-HSVtk* mice following GCV exposure and removal *in vivo*. (**a**,**b**) *Stra8* mRNA levels (**a**) and percentage of Stra8-immunopositive seminiferous tubules (**b**) in testes of adult *pStra8-Gfp* or *pStra8-HSVtk* mice following 28 days of vehicle (PBS) or GCV (1 mg kg^−1^) exposure, or 21 days after cessation of GCV treatment (Post-GCV). Data represent the mean ± s.e.m. (*n* = 3 mice per group; *P < 0.05). (**c,d**) Representative Stra8 protein expression in (**c**; *brown*, against a *blue* hematoxylin counterstain; left panels, ×10 with asterisks marking Stra8-immunopositive tubules; right panels, ×40), and histological appearance of (**d**; left panels, ×4; right panels, ×40), testes of adult *pStra8-Gfp* mice following 28 days of GCV exposure (+GCV, 1 mg kg^−1^), or in testes of adult *pStra8-HSVtk* mice following 28 days GCV exposure (+GCV) or 21 days after cessation of GCV treatment (Post-GCV). Scale bars, 50-μm.

As a final control for the specificity of suicide gene targeting prior to embarking on studies of adult ovaries, we tested our system in adult *pStra8-HSVtk* male mice since it is well documented that SSCs support spermatogenesis through Stra8-mediated meiotic activation^66, 67^. Using age-matched *pStra8-Gfp* male mice treated in parallel to monitor potential toxicity of high doses of GCV to spermatogenesis in the absence of *HSVtk* expression^74^, we determined that administration of GCV at 1 mg kg^−1^ each day for 28 days decreased *Stra8* expression in testes of *pStra8-HSVtk* male mice without affecting *Stra8* expression in *pStra8-Gfp* control males (Fig. 2a). Immunohistochemical and histological evaluations revealed that testes from GCV-treated *pStra8-HSVtk* male mice showed a significant reduction in the percentage of Stra8-positive seminiferous tubules (Fig. 2b,c) along with decreased cellularity and disrupted spermatogenesis (Fig. 2c). However, these effects were not observed in testes of *pStra8-Gfp* male mice exposed to GCV in parallel (Fig. 2b,c). Over a 21-day recovery period following cessation of GCV treatment, *pStra8-HSVtk* male mice regenerated *Stra8*-expressing germ cells (Fig. 2b,c) to support a resumption of spermatogenesis and a return to normal testicular morphology (Fig. 2c). These findings showed that GSC progeny could be effectively targeted for ablation in a temporally controlled and reversible manner *in vivo* using this *pStra8-HSVtk* suicide gene-based approach, and that the GSCs themselves remain competent to continue support of new gamete formation after the cessation of pro-drug treatment.

### Oocyte regeneration in ovaries of adult suicide gene transgenic mice

**Figure 3:**
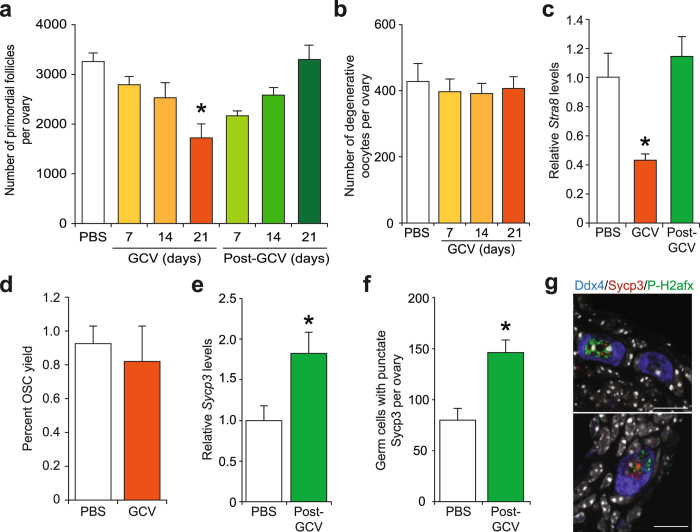
Dynamics of oocyte reserve depletion and regeneration in adult female *pStra8-HSVtk* mice following GCV exposure and removal *in vivo*. (**a**) Number of primordial oocyte-containing follicles in ovaries of young adult *pStra8-HSVtk* mice following 21 days of treatment with vehicle (PBS) or GCV (10 mg kg^−1^; completed on postpartum day 69), or 21 days after cessation of GCV treatment (Post-GCV, completed on postpartum day 90). Data represent the mean ± s.e.m. (*n* = 4–6 mice per group; *P < 0.05). (**b**) Number of atretic (dying, dead) oocytes in ovaries of young adult *pStra8-HSVtk* mice treated with vehicle (PBS) or GCV (10 mg kg^−1^) for up to 21 days (mean ± s.e.m., *n* = 4–6 mice per group). (**c**) *Stra8* mRNA levels (normalized against *β-actin* mRNA levels) in ovaries contralateral to those used to derive the oocyte counts shown in panel a (mean ± s.e.m., *n* = 4–6 mice per group; *P < 0.05). (**d**) Yield of FACS-purified OSCs (percent of total viable cells sorted) from ovaries of young adult *pStra8-HSVtk* mice following 21 days of treatment with vehicle (PBS) or GCV (10 mg kg^−1^). Data represent the mean ± s.e.m., *n* = 3 mice per group. (**e**) *Sycp3* expression in ovaries of young adult *pStra8-HSVtk* mice 21 days after ceasing treatment with vehicle (PBS) or GCV (10 mg kg^−1^). Data represent the mean ± s.e.m., *n* = 3–4 mice per group; *P < 0.05. (**f**) Number of Ddx4-positive germ cells with punctate nuclear localization of Sycp3 protein in ovaries contralateral to those used for panel e (mean ± s.e.m., *n* = 3 mice per group; *P < 0.05). (**g**) Immunofluorescence images of Ddx4-positive germ cells (*blue*) co-expressing Sycp3 (*red*) and Ser^139^-phospho-H2afx (*green*) in ovaries of young adult *pStra8-HSVtk* mice 21 days after ceasing a 3-week course of GCV treatment (10 mg kg^−1^). DAPI nuclear stain, *white*; scale bars, 10-μm.

With these controls in place, we next tested if the reversible gametogenic failure observed in *pStra8-HSVtk* male mice following GCV exposure and removal (Fig. 2) also occurs in adult *pStra8-HSVtk* female mice treated in a similar manner. As a baseline for study and subsequent data interpretation, oocyte-containing follicle numbers were determined to be comparable in 1.5-month-old (day 48 postpartum) wild type and *pStra8*-*Gfp* female mice, and treatment of females of either control genotype with GCV for 21 days had no effect on ovarian *Stra8* expression or numbers of existing oocytes (Supplementary Fig. S4). Follicle numbers in 1.5-month-old *pStra8-HSVtk* female mice exposed to vehicle (Fig. 3a) were likewise comparable to those of wild type and *pStra8-Gfp* control females (Supplementary Fig. S4). These data collectively confirmed that the existing oocyte reserve is unaffected by either pro-drug administration in the absence of *HSVtk* expression or, conversely, *HSVtk* expression in the absence of pro-drug administration. Following daily administration of GCV for 21 days to 1.5-month-old *pStra8-HSVtk* mice, the ovaries appeared histologically normal (Supplementary Fig. S5), and we observed no significant changes in the number of degenerative (atretic) oocytes during the entire GCV treatment course (Fig. 3b). This was important since it documented a complete absence of any ‘off-target’ oocyte death in response to GCV exposure in this *pStra8-HSVtk* transgenic model.

However, ovarian *Stra8* expression (Fig. 3c) and primordial oocyte-containing follicle numbers (Fig. 3a) were significantly lower in 1.5-month-old *pStra8-HSVtk* mice exposed to GCV for 21 days compared to vehicle injected *pStra8-HSVtk* controls. Quantitative analysis indicated that ovaries of GCV-treated *pStra8-HSVtk* mice contained almost 1,600 fewer primordial oocytes compared to age-matched *pStra8-HSVtk* females treated with vehicle in parallel (Fig. 3a), and this was not a result of existing oocyte depletion to due increased oocyte death (Fig. 3b). To test if this oogenic failure phenotype was reversible, 1.5-month-old *pStra8-HSVtk* female mice were treated with GCV for 21 days (starting on postpartum day 48) and then maintained for 21 additional days after ceasing GCV exposure (post-GCV recovery phase ending on postpartum day 90). A spontaneous return of ovarian *Stra8* expression (Fig. 3c) and complete regeneration of the primordial oocyte population (Fig. 3a) occurred over this 21-day recovery period. Since OSCs freshly isolated from adult ovaries do not express *Stra8* (Fig. 1d and Supplementary Fig. S3; see also ref. 18), these cells should be unaffected by GCV exposure. This was verified by our findings of a comparable yield of OSCs from ovaries of 1.5-month-old *pStra8-HSVtk* female mice treated for 21 days with vehicle or GCV (Fig. 3d). Once re-established, the regenerated oocyte pool in *pStra8-HSVtk* females remained stable such that no differences were observed in numbers of primordial or growing follicles between vehicle- and GCV-treated mice 3 months after cessation of GCV exposure (Supplementary Fig. S6a). In addition, long-term fertility and fecundity parameters were also comparable in *pStra8-HSVtk* female mice treated with vehicle versus GCV (Supplementary Fig. S6b).

If OSC differentiation is involved in the burst of *de-novo* oogenesis detected in adult ovaries during the post-GCV oogenic recovery period (Fig. 3a), evidence of active germ cell meiosis should be apparent. Therefore, we next evaluated ovaries of *pStra8-HSVtk* mice for expression of synaptonemal complex protein 3 (Sycp3), which is widely known to mark germ cells entering prophase-I of meiosis^75^. During the post-GCV recovery phase, we observed a significant increase in both ovarian *Sycp3* expression (Fig. 3e) and numbers of Ddx4-positive germ cells exhibiting a pattern of punctate nuclear Sycp3 protein expression (Fig. 3f,g). These Ddx4-Sycp3 dual-positive ovarian cells also expressed Ser^139^-phosphorylated H2A histone family member X (P-H2afx) (Fig. 3g), which is known to localize to DNA double-strand breaks in germ cells during the early stages of meiotic prophase^76^. This evidence of active meiotic differentiation of germ cells, coupled with the reversible oogenic failure phenotype observed in response to specific targeting of germ cells activating *Stra8* expression in *pStra8-HSVtk* transgenic females following GCV exposure and removal (Fig. 3a), collectively support that an important role exists for *de-novo* oogenesis in maintenance of the adult oocyte reserve.

### Oocytes formed during adult life generate offspring

**Figure 4:**
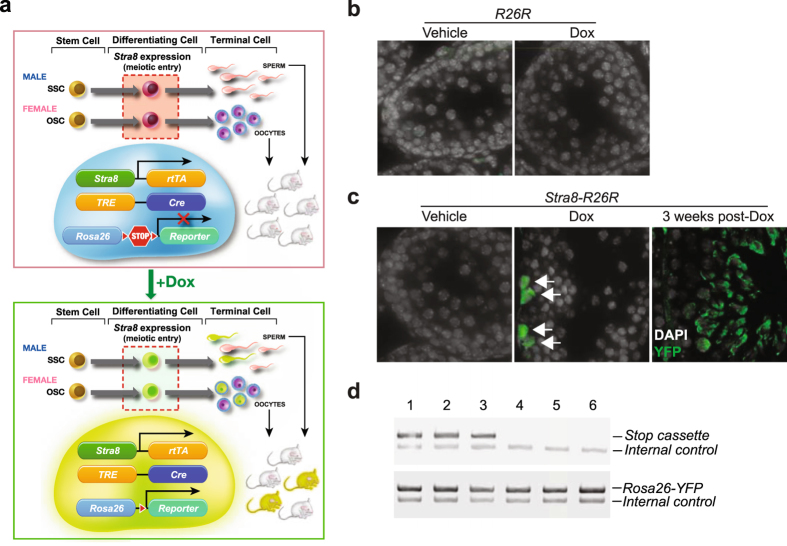
Genetic fate mapping strategy and its application to tracing SSC progeny after meiotic differentiation. (**a**) Schematic depiction of the genetic strategy used to permanently ‘mark’ GSCs committing to meiosis in adult gonads in an inducible manner. (**b**) Absence of YFP expression in testes of control (*R26R* or ‘promoterless’ *TRE-Cre*;*Rosa26-Stop-Yfp*) transgenic males induced with Dox (1 mg kg^−1^) for 28 days (DAPI nuclear stain, *white*). (**c**) Detection of YFP expression (*green*) in spermatogonia lining the inner surface of the basement membrane of the seminiferous tubules in *pStra8-R26R* male mice after 28 days of induction with Dox (1 mg kg^−1^), and progression of ‘marked’ germ cells to spermatids 3 weeks later (DAPI nuclear stain, *white*). (**d**) Genotype analysis of first-generation offspring sired by *pStra8-R26R* males induced with Dox (1 mg kg^−1^) for 28 days before housing with wild type females, showing the presence of babies derived from fertilization of eggs by both non-recombined (*Stop* cassette intact; offspring 1–3) and recombined (*Stop* cassette excised; offspring 4–6) spermatozoa. Complete (uncropped) PCR gels for each target sequence amplified are shown in Supplementary Fig. S7.

To next assess if oocytes generated during adulthood contribute directly to fertility, we replaced the *Gfp* coding sequence in our *pStra8-Gfp* construct with *reverse tetracycline-controlled transactivator* (*rtTA*) and generated a knock-in transgenic *pStra8-rtTA* mouse line. We then introduced two additional alleles into *pStra8-rtTA* mice: 1) a *tetracycline responsive element* (*TRE*)*-driven Cre recombinase* (*TRE-Cre*) construct, and 2) a *Rosa26-Stop-Yfp* reporter construct containing a floxed *phosphoglycerate kinase 1* (*Pgk*) promoter-driven *neomycin phosphotransferase* (*Npt*) cassette that prevents *Rosa26*-driven transcription of the downstream *Yfp* coding sequence in the absence of Cre recombinase. This approach generated a fluorescent reporter mouse line (*pStra8-R26R*), in which activation of the *Stra8* promoter drives expression of rtTA, leading to Cre recombinase activation and, subsequently, *Rosa26-*driven *Yfp* expression through excision of the floxed *Pgk*-*Npt* (*Stop*) sequence only in the presence of doxycycline (Dox). As a result, any germ cells that activate meiosis through *Stra8* during a window of Dox exposure will become permanently ‘marked’, thus enabling us to trace the fate of these cells *in vivo* under normal physiological conditions (Fig. 4a).

As a positive control, we first performed lineage marking of *Stra8*-expressing cells in *pStra8-R26R* male mice following Dox treatment. We observed YFP expression in germ cells along the basement membrane of the seminiferous tubules in the testes (Fig. 4b,c), where endogenous Stra8-expressing cells are known to be located (Fig. 2c). Three weeks after a single Dox induction, differentiated spermatids within the seminiferous tubules were found to be YFP-positive (Fig. 4c). In mating trials with wild type female mice, males induced with Dox sired pups carrying the recombined *Rosa26-Yfp* allele (Fig. 4d and Supplementary Fig. S7), confirming utility of this system to fate-map germ cells undergoing meiotic differentiation *in vivo*. In turn, as negative controls we did not observe YFP expression in ovaries of either *pStra8-R26R* female mice treated with vehicle (*n* = 5 mice) or R26R (‘promoterless’ *TRE-Cre*;*Rosa26-Yfp*) female mice induced with Dox for 21 days (*n* = 5 mice) (Supplementary Fig. S8).

**Figure 5:**
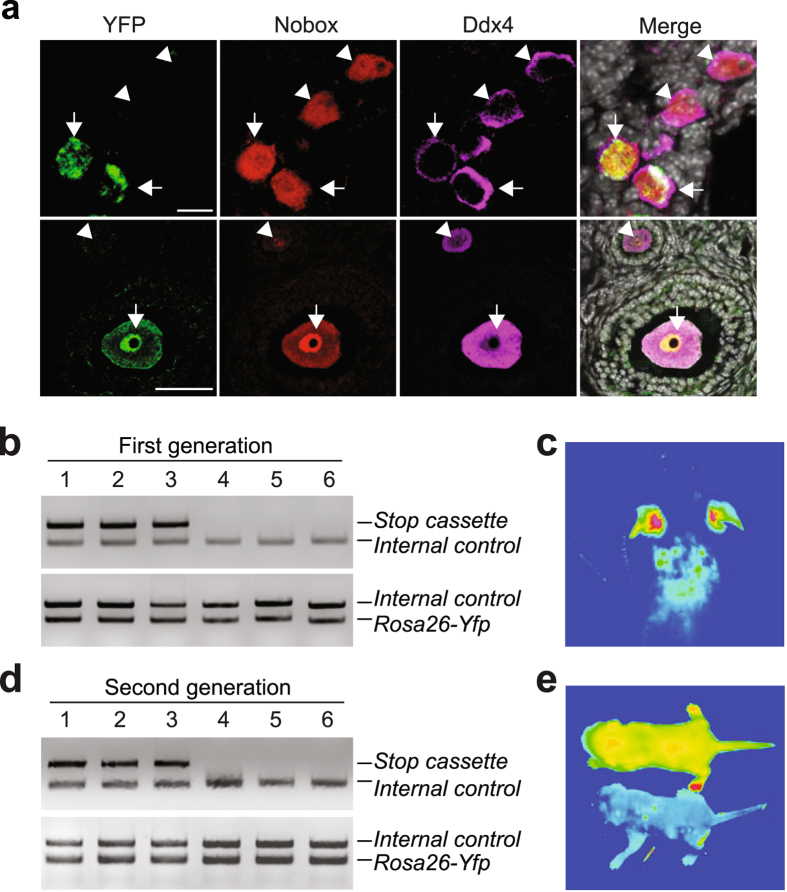
Genetic fate mapping of oocytes generated during adulthood to the generation of viable offspring. (**a**) Representative expression analysis of YFP (*green*; recombined and activated reporter), Ddx4 (*red*; germ cell marker) and Nobox (*purple*; oocyte marker) proteins in ovaries of young adult *pStra8-R26R* mice after 21 days of induction with Dox (2 mg ml^−1^); DAPI nuclear stain in the merge panel, *white*. Note the proximity of oocytes newly formed during the Dox induction phase (YFP^+^/Ddx4^+^/Nobox^+^, *arrows*) adjacent to oocytes pre-existing before the start of Dox induction (YFP^–^/Ddx4^+^/Nobox^+^, *arrowheads*). (**b**) Genotype analysis of first-generation (F_1_) offspring delivered by *pStra8-R26R* females induced with Dox (10 mg kg^−1^) for 21 days before housing with wild type males, confirming the presence of babies derived from natural fertilization of both pre-existing oocytes (non-recombined, *Stop* cassette intact; offspring 1–3) and oocytes formed during the induction period (recombined, *Stop* cassette excised; offspring 4–6) in adult ovaries. Complete (uncropped) PCR gels for each target sequence amplified are shown in Supplementary Fig. S9a. (**c**) Live fluorescence image of a representative F_1_-generation mouse identified by genotyping (**b**) as being derived from fertilization of an oocyte formed during adulthood in a Dox-induced *pStra8-R26R* female. (**d,e**) Natural mating of recombined F_1_ generation female offspring with wild type males reveals germline transmission of the recombined *Rosa26-Yfp* allele to F_2_ generation offspring (**d**; complete (uncropped) PCR gels for each target sequence amplified are shown in Supplementary Fig. S9b), which was confirmed by live fluorescence imaging of ubiquitous YFP expression (**e**; a representative F_2_-generation pup of each genotype is shown for comparative purposes).

However, we detected YFP-positive oocytes, enclosed within follicles and co-expressing the immature oocyte transcription factor, Nobox, in ovaries of adult *pStra8-R26R* female mice treated with Dox for 21 days (Fig. 5a). Marked oocytes formed during Dox induction were often located adjacent to unlabeled (pre-existing) oocytes, which were also positive for Nobox and contained within follicles (Fig. 5a). To test if marked oocytes formed during adulthood are fully functional, female *pStra8-R26R* mice were induced for 21 days with Dox and then housed with wild type males. Consistent with formation of a mosaic oocyte pool following induction of the reporter (Fig. 5a), Dox-induced females gave birth to mosaic litters composed of offspring lacking (YFP-negative), and offspring exhibiting (YFP-positive), recombination at the *Rosa26-Stop-Yfp* locus (Fig. 5b and Supplementary Fig. 9a). These data essentially mirrored those obtained from parallel studies of *pStra8-R26R* male mice used as a positive control (Fig. 4d and Supplementary Fig. S7). Live-imaging revealed widespread YFP expression in recombined offspring, which grew to adulthood without issue (Fig. 5c). Mating of first-generation (F_1_) female offspring carrying the recombined *Rosa26-Yfp* allele with wild type males confirmed germline transmission of the recombined reporter gene to F_2_ offspring (Fig. 5d and Supplementary Fig. S9b), which by live imaging also exhibited widespread YFP expression (Fig. 5e). These results demonstrated that oocytes newly formed in adult ovaries *in vivo* contribute directly to natural female fertility under physiological conditions.

### OSC function in adult ovaries declines with age

**Figure 6:**
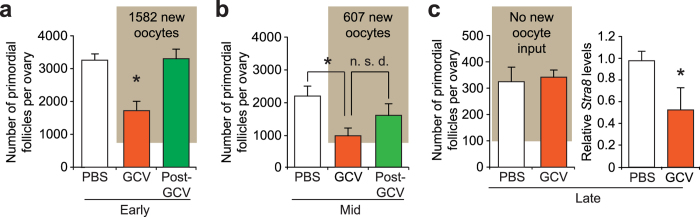
Aging female mice show a progressively diminished potential for *de-novo* oogenesis. (**a,b**) Comparison of changes in the primordial follicle reserve in ovaries of *pStra8-HSVtk* mice in which GCV treatment (10 mg kg^−1^) was initiated on postpartum day 48 with the post-GCV regenerative phase occurring between 2–3 months of age (**a**; early reproductive life, Early), or on postpartum day 134 with the post-GCV regenerative phase occurring between 5–6 months of age (**b**; mid-reproductive life, Mid). Data represent the mean ± s.e.m., *n* = 3–6 mice per group; *P < 0.05; n.s.d., not significantly different. (**c**) Lack of effect of 21 days of GCV treatment (10 mg kg^−1^) on primordial follicle numbers in *pStra8-HSVtk* mice treated between 10–11 months of age (late reproductive life, Late); note that while oocyte numbers in *pStra8-HSVtk* females at advanced (late) reproductive age were unaffected by 21 days of GCV treatment, endogenous ovarian *Stra8* expression remained sensitive to GCV exposure in these mice (mean ± s.e.m., *n* = 4–5 mice per group; *P < 0.05).

Past studies with mice have shown that the primordial oocyte pool, once established during the early juvenile period, remains remarkably stable during the first 3 months of life despite a high daily rate of exit (growth activation) and atresia, but then shows significant depletion as the females reach 5–6 months of age^6, 10, 11^. To determine if progressive loss of the oocyte reserve after 3 months of age is due, at least in part, to a declining capacity for oocyte renewal, we compared the magnitude of the post-GCV oogenic recovery response in *pStra8-HSVtk* female mice during early, mid- and late reproductive ages (Fig. 6a,b). After 3 weeks of GCV exposure from postpartum days 134 to 155 (mid-reproductive life), primordial oocyte numbers in *pStra8-HSVtk* females was significantly lower compared to those of vehicle-injected controls (Fig. 6b), in a manner that paralleled the response of *pStra8-HSVtk* females when GCV was administered during early reproductive life from postpartum days 48 to 69 (Fig. 6a). However, compared to the robust post-GCV oogenic response (1,582 new oocytes) observed in females from postpartum days 69 to 90 (early reproductive life; Fig. 6a), the post-GCV oogenic response spanning postpartum days 155 to 176 (mid-reproductive life) was severely blunted (only 607 new oocytes). In fact, in *pStra8-HSVtk* females during mid-reproductive life, the size of the primordial oocyte pool after 21 days of post-GCV recovery was not significantly different from the size of the pool when GCV treatment was terminated (Fig. 6b). By 10 months of age (late reproductive life), the primordial oocyte pool in vehicle-treated *pStra8-HSVtk* females was severely diminished due to advanced maternal age, and 21 days of GCV exposure no longer elicited any change in the size of the oocyte reserve (Fig. 6c). This outcome provided not only evidence of an absence of active oogenesis by this time in life but also additional evidence of a complete lack of off-target killing of oocytes by GCV (see also Fig. 3b). Interestingly, however, GCV treatment still elicited a decrease in ovarian *Stra8* expression in females at 10 months of age (Fig. 6c). We interpreted this to indicate that OSCs were still present and attempting Stra8-mediated differentiation even at this advanced maternal age, but the selective ablation of *Stra8*-expressing cells by GCV treatment effectively ‘removed’ the levels of *Stra8* mRNA transcript normally contributed by these cells to the overall pool of ovarian mRNA analyzed.

**Figure 7:**
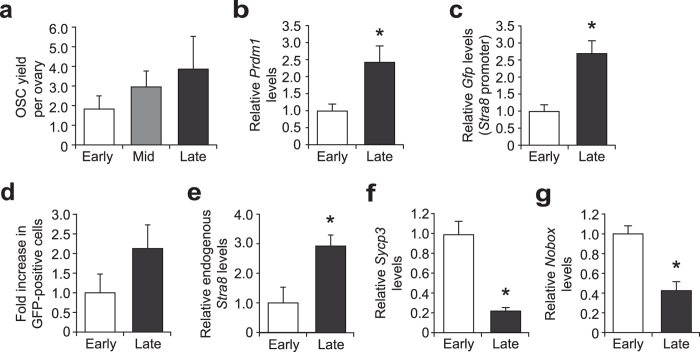
Evidence that the diminished oogenic potential in reproductively aged females is not due to an absence of OSCs in the ovaries. (**a**) Yield of FACS-purified OSCs from ovaries of female mice at early, mid- and late reproductive ages (mean ± s.e.m., *n* = 3–4 mice per group). (**b**,**c**) Quantitative analysis of *Prdm1* and *Stra8* promoter-driven *Gfp* mRNA levels in ovaries of *pStra8-Gfp* mice at early and late reproductive ages (mean ± s.e.m., *n* = 6 mice per group; *P < 0.05). (**d**) Yield of *Stra8* promoter-driven GFP-positive cells from ovaries of *pStra8-Gfp* mice at early and late reproductive ages (mean ± s.e.m., *n* = 6–8 mice per group with 2 mice for each sample and 3–4 samples at each age). (**e**–**g**) Quantitative analysis of endogenous *Stra8*, *Sycp3* and *Nobox* mRNA levels in ovaries of *pStra8-Gfp* mice at early and late reproductive ages (mean ± s.e.m., *n* = 6 mice per group; *P < 0.05).

Consistent with this, and with recent observations from studies of mouse and human ovarian tissues^77, 78^, we found that the yield of OSCs from adult ovaries increased slightly with advancing maternal age (Fig. 7a). This observation, coupled with the increased levels of ovarian expression of the primitive germ cell marker, *PR domain containing 1 with ZNF domain* (*Prdm1*), in 10–11-month-old females (Fig. 7b), collectively indicated that the loss of oogenic potential with age (Fig. 6a–c) is probably due more to impaired OSC function rather than an aging-dependent disappearance of OSCs from the gonads. In ovaries of *pStra8-Gfp* mice at 10–11 months of age, we also detected an age-related increase in *Stra8* promoter-driven *Gfp* expression (Fig. 7c), increased numbers of GFP-expressing cells (Fig. 7d), and increased expression levels of the endogenous *Stra8* gene (Fig. 7e). However, expression of *Sycp3* dropped precipitously in ovaries at late reproductive ages (Fig. 7f), along with an expected decline in expression of the immature oocyte marker, *Nobox* (Fig. 7g). Thus, OSCs are still present, and likely attempting meiotic differentiation, in ovaries at advanced reproductive ages; however, meiotic progression after *Stra8* activation apparently then fails, perhaps due to waning Sycp3 availability.

## Discussion

Through use of two widely accepted genetic approaches for the *in-vivo* study of adult stem cell function, here we show that adult mouse ovaries actively generate new oocytes that are subsequently used for ovulation, fertilization and the generation of viable offspring. As important as these observations are, the current lack of a gene promoter that is exclusively expressed in OSCs, but not in differentiating premeiotic germ cells or oocytes, does not enable unequivocal identification of OSCs as the cells responsible for supporting postnatal oogenesis. However, the independent verification of the presence of OSCs in the ovaries of a growing number of mammalian species^10, 12–35, 46, 78^, the ability of OSCs to generate new oocytes and functionally competent eggs in adult females^12, 15, 16, 20, 21, 25, 32, 34, 35^ (Supplementary Fig. S1), and experimental evidence that resident multi-potent stem cells in adult ovaries are unable to generate oocytes^79^, collectively support that OSCs are the most logical and likely source of new oocytes formed during adulthood. With that said, a few recent studies have concluded based on negative data that OSCs do not exist in postnatal mouse or human ovaries^36, 37, 80^. However, a detailed re-assessment of the first of these studies using the experimental mouse models and protocols reported in the original paper^36^ uncovered several design weaknesses and a lack of important controls in the approaches employed to reach the conclusion that OSCs do not exist; when these were accounted for and corrected, OSCs were purified without issue^26^. These latter observations were verified and extended by recent work from a different laboratory, which also showed that OSCs could be purified by FACS from ovaries of germline-specific fluorescent gene reporter mice; further, the purified OSCs gave rise to offspring following intraovarian transplantation into wild type female recipients^34^. Design concerns with other studies questioning the existence of OSCs and postnatal oogenesis^37, 80^ have also been identified^81^ and addressed experimentally^30^. Notably, two very recent studies have provided the first insights into the post-transcriptional and epigenetic determinants of unipotency and other germline properties in OSCs, as well as the identity of factors that maintain OSCs in an undifferentiated state^82, 83^. Additionally, by tracing individual transplanted OCSs expressing EGFP, Wu and colleagues also recently mapped the *in-vivo* differentiation of these cells into new oocytes following intraovarian delivery, as well as the high similarity in gene expression networks in follicles containing oocytes formed from transplanted OSCs versus endogenous (pre-existing) wild type follicles^35^.

In considering our first approach, clear interpretation of data derived from use of suicide gene technology is dependent on several variables. First, specificity of the gene promoter used to target the suicide gene to a desired cell type is critical. In full agreement with a large volume of existing work reporting on the specificity of *Stra8* expression in pre-meiotic germ cells in mice^18, 63–68, 70^, our in-depth assessment of the GFP-positive cell fraction isolated from ovaries of *pStra8-Gfp* transgenic mice (Fig. 1d,e) demonstrated that the 1.4-kb *Stra8* promoter fragment used for suicide gene targeting is indeed restricted to germ cells (positive for *Pou5f1*, *Dppa3* and *Ddx4*) that are committing to meiosis (positive for *Stra8*, at the level of both promoter activity and endogenous gene expression) but have not yet differentiated into new oocytes (negative for *Nobox*, *Sohlh1* and *Zp3*). In addition, the reversible spermatogenic failure observed following GCV treatment and removal in *pStra8-HSVtk* male mice (Fig. 2) – a model in which GSC support of adult gametogenesis is universally accepted^1, 2^, further indicates that use of this specific *Stra8* promoter fragment to drive GCV-sensitive *HSVtk* expression meets the requirements for targeting early germ cells initiating meiotic commitment. Second, the endpoint examined must be unaffected by the suicide gene in the absence of its pro-drug and by pro-drug exposure in the absence of its suicide gene. In-depth assessment of oocyte dynamics and endogenous *Stra8* expression in wild type, *pStra8-Gfp* and *pStra8-HSVtk* mice exposed to vehicle or GCV (Fig. 5a and Supplementary Fig. S8) satisfied this requirement. Finally, the readout must reflect the endpoint activity of the cells targeted for ablation. In our case, alterations in OSC daughter cell differentiation into oocytes is the most biologically plausible explanation for the loss and, without question, the subsequent spontaneous regeneration of the primordial oocyte-containing follicle pool in *pStra8-HSVtk* females after GCV exposure and removal, respectively (Fig. 3a). Further to this point, very recent studies of adult female rats exposed to agents known to be highly toxic to oocytes showed that the initial reduction in primordial follicle numbers observed shortly after drug treatment was followed by a robust period of oogenic recovery, leading to regeneration of the primordial follicle pool^84^.

It is also worth noting that a ‘bystander effect’ caused by release of cytotoxic metabolites from suicide gene-expressing cells, which may then kill surrounding wild type (off-target) cells, has been raised as a confounding issue in the use of suicide gene systems^85, 86^. However, bystander killing effects have not been observed in mouse models designed to characterize the role of a specific cell type *in vivo* ^47, 48, 87, 88^. Further, a clear absence of any change in the incidence of oocyte death in *pStra8-HSVtk* females during the entire 21-day course of GCV exposure (Fig. 3b) establishes two important principles: 1) the smaller oocyte pool detected in *pStra8-HSVtk* females after 21 days of GCV treatment does not result from non-specific or off-target killing of existing oocytes during GCV exposure, a point further reinforced by the complete lack of effect of GCV treatment on the oocyte pool in females at 10 months of age (Fig. 6c); and, 2) the *Stra8* promoter fragment used to produce our transgenic lines is not active in existing oocytes. The latter point is also fully consistent with an absence of either *Stra8* promoter driven *Gfp* expression or endogenous *Stra8* mRNA in oocytes of *pStra8-Gfp* females (Fig. 1d), as well as an absence of any well-accepted oocyte markers in the GFP-expressing cell fraction purified from ovaries of *pStra8-Gfp* females by FACS (Fig. 1d). Even in the unlikely case that undetected bystander killing is occurring in this model and the existing oocyte reserve is somehow negatively affected by GCV in a manner we cannot discern, the spontaneous and complete regeneration of primordial oocyte numbers back to pre-treatment levels after ceasing GCV exposure (Fig. 3a) provides unequivocal evidence for the occurrence of active oogenesis and folliculogenesis in adult ovaries. This conclusion agrees with recent studies of *Pou5f1*-*MerCreMer* transgenic mice crossed with *R26R-enhanced yellow fluorescent protein* transgenic mice to establish a tamoxifen-inducible system for labeling *Pou5f1*-expressing cells in postnatal ovaries^89^. Although use of the *Pou5f1* gene promoter to drive reporter expression for fate mapping in the ovary is complicated by expression of *Pou5f1* in cell types aside from OSCs, including oocytes^58^ and resident multi-potent stem cells^79^, evidence for the occurrence of germ cell proliferation, meiotic entry and *de-novo* replenishment of the primordial follicle pool in adult mouse ovaries was provided^89^. Notably, our assessment of meiotic activation and progression during the post-GCV recovery phase (Fig. 3e–g) in turn independently verifies the recent report from Gou and colleagues regarding their detection of germ cell meiotic entry in adult mouse ovaries using *Pou5f1*-*MerCreMer* transgenic mice^89^.

As compelling as we believe these observations are using suicide gene technology, the limitation with this approach is uncertainty over what, if anything, oocytes formed during adulthood do. By extending this work through parallel use of genetic lineage tracing from the premeiotic germ cell stage, we have shown that active *Stra8*-mediated germline differentiation is not only required for maintenance of oocyte numbers in female mice during early to mid-adult life (Figs. 3 and 6), but also that oocytes formed during adulthood contribute directly to the pool of eggs used for the generation of offspring in natural mating trials (Fig. 5). Such documentation of a physiological role for postnatal oogenesis in mammals provides an impetus to reconsider current thinking on many aspects of female reproductive biology, including a reassessment of underlying events responsible for ovarian failure with age. For example, in mammalian testes, SSCs are known to persist into advanced age, even after spermatogenic failure has occurred^90, 91^. When SSCs from aged atrophic testes are transplanted into young adult testes, the cells resume spermatogenesis^90^. Likewise, past studies have shown that primordial oocyte formation in aged mouse ovaries also resumes when the tissue is grafted into a young adult ovarian environment^77^. In considering this prior report with our findings presented herein, depletion of the oocyte pool in female mammals with age, which appears to result from a combination of oocyte loss through growth activation followed by ovulation or atresia coupled with a progressive decline in new oocyte input, might be amenable to prevention or even reversal. Another shift in thinking revolves around the fact that the postnatal oocyte reserve is not a finite entity at birth as previously believed^5^. This change in thinking, coupled with recent reports that a comparable population of mitotically-active germ cells not only exists in adult human ovaries^16, 17, 22, 27–29, 31, 33^ but can also differentiate into IVD-oocytes in culture and into immature oocytes in human ovarian tissue xenografts *in vivo* ^16, 17, 22, 27, 33^, provide a solid foundation on which to further explore the potential of OSC-based technologies for management of ovarian function and female infertility^78, 81, 92^.

## Methods

### Animals and treatments

Wild type C57BL/6 mice were from Charles River Laboratories, *TRE-Cre* (strain: Tg(tetO-cre)1Jaw/J; stock number: 006224), *Rosa26-Stop-Yfp* (strain: B6.129 × 1-*Gt*(*ROSA*)*26Sor* ^*tm1*(*EYFP*)*Cos*^/J; stock number: 006148) and *OG2* (strain: B6; CBA-Tg(Pou5f1-EGFP)2Mnn/J; stock number: 004654) mice were from the Jackson Laboratory, and *pStra8-Gfp* mice were generated as described^18^. Transgenic mice with *HSVtk* or *rtTA* driven by the 1.4-kb *Stra8* promoter fragment were generated by replacing the GFP-coding sequence in the *pStra8-Gfp* construct^18^ with cDNA encoding GFP-fused HSVtk (provided by J. Galipeau) or encoding rtTA, and the constructs were then sent to Genoway for generation of the indicated knock-in transgenic lines^18^. For comparative studies, wild type and transgenic siblings from breeding colonies were used in parallel to rule out any potential effect of background strain on the outcomes. For treatments, GCV (Roche) was dissolved in sterile water at 10 mg ml^−1^, and then diluted in sterile 1X-concentrated PBS for daily dosing (males: 1 mg kg^−1^ for 28 days; females: 10 mg kg^−1^ for 21 days). Treatment protocols were based on prior studies of suicide gene-based ablation of somatic cells^47–49, 87, 88^, and on empirical testing using *pStra8-Gfp* male and female mice as negative controls for GCV dosing. Doxycycline was administered for 21 days via the drinking water at a concentration of 2 mg ml^−1^. Where indicated, whole body fluorescence imaging of live mice was performed using a Nikon OVA110 imaging system. All animal studies were approved by the appropriate institutional animal care and use committees at Northeastern University and Massachusetts General Hospital, and all methods used in this study were performed in accordance with all relevant institutional guidelines and regulations.

### Germ cell isolation, culture and IVD-oocyte formation

For most experiments (except as indicated below), OSCs were isolated from ovaries of young adult mice (2–3 months of age) by FACS using a C-terminal DDX4-specific antibody (ab13840, Abcam). The cells were analyzed immediately or established in culture without somatic feeder cells, as described^16, 17, 46, 93^. Purified mouse OSCs propagated under these conditions spontaneously differentiate into IVD-oocytes for up to 72 h after passage until confluence is regained, and the number of IVD-oocytes generated by a fixed number of OSCs seeded per well remains relatively constant over successive passages^16, 17, 19^. Between passages 32–40, OSCs were transfected with the desired plasmids (*pStra8-HSVtk* or *pStra8-Gfp*, each containing a *neomycin resistance* gene) using Lipofectamine 2000 (Invitrogen) and then selected by G418 (Geneticin, Cellgro) over 2 weeks. Cells were then maintained in G418 for all experiments, and the number of IVD-oocytes generated and released into the medium after treatment with vehicle or GCV (2 μM) was then determined by direct visual counts under a microscope^16, 17, 19^. In other experiments, GFP-positive cells in ovaries of *pStra8-Gfp* transgenic female mice were quantitated and then isolated by FACS for gene expression profiling.

### Intraovarian OSC transplantation

Mouse OSCs, isolated from ovaries of young adult *OG2* transgenic female mice as described above, were injected directly into each ovary (~1 × 10^4^ viable cells per injection) of four recipient wild type C57BL/6 female mice at 2 months of age, as detailed previously^16^. After a one-week recovery period, adult wild type males were introduced into the cages with the transplanted females for mating trials over a subsequent 4-month period. All offspring were genotyped for the absence or presence of the *OG2* transgene.

### Oocyte counts

Young adult mouse ovaries were fixed, serially sectioned and processed for histomorphometry-based quantification of the number of healthy or degenerative (atretic) oocyte-containing follicles at the indicated stages of development, as detailed^10, 94^. All samples were assessed in a completely blinded fashion, and reproducibility was independently confirmed in a blinded fashion by two other observers. In all cases, variation in counts between observers was less than 7% (Supplementary Fig. S10). As an additional verification of the counting method employed, serially sectioned mouse ovaries were processed for immunohistochemical detection of the oocyte-specific marker, Nobox (ref. 71), after which the number of Nobox-positive oocytes contained within immature follicles was quantified for each ovary. These counts, and those obtained by direct visual assessment (oocyte morphology through histology), were then compared and found to produce similar data sets (Supplementary Fig. S10).

### Gene expression analysis

Total RNA was extracted using Tri-Reagent (Sigma-Aldrich) and reverse transcribed (Superscript III; Invitrogen) using oligo-dT primers. For some experiments, amplification of target gene sequences was performed by conventional PCR to assess for the absence or presence of *Gfp*, *Pou5f1*, *Ddx4*, *Prdm1*, *Dppa3*, *Ifitm3*, *Stra8*, *Nobox*, *Sohlh1* or *Zp3* expression, as well as of *β-actin* expression as a sample loading control (see Supplementary Table S1 for details). All products were sequenced to confirm identity. For quantitative analysis of mRNA levels, real-time PCR was performed using a Cepheid Smart Cycler II. For *Stra8* expression (normalized against *β-actin* mRNA levels), primers for *Stra8* (FAM-labeled D-LUX^TM^ Pre-designed Gene Expression Assays, MLUX3312362) and *β-actin* (FAM-labeled certified LUX^TM^ Primer Set for Mouse/Rat *β-actin*, 101M-01) were obtained from Invitrogen. For assessment of *Sycp3*, *Prdm1*, *Gfp* and *Nobox* expression (normalized against *β-actin* mRNA levels), SYBR-based quantitative PCR was conducted by using BioRad SsoAdvanced™ Universal SYBR^®^ Green Supermix along with gene-specific primers (Supplementary Table S1).

### Genotyping of *pStra8-R26R* mice

Recombination at the *Rosa26-Stop-Yfp* locus was confirmed by genotyping offspring for the presence of the *Yfp* coding sequence along with excision of the floxed *Pgk-Npt* (*Stop*) cassette using primers specific for *Yfp* and *Npt*, respectively (Supplementary Table S1). As an internal control for PCR quality, primer sets against *intestinal fatty acid binding protein 2* (*Fabpi*) were included during PCR for detection of *Npt* (*Fabpi* short, 194 bp) and of *Yfp* (*Fabpi* long, 466 bp) (Supplementary Table S1).

### Immunofluorescence (IF) and immunohistochemistry (IHC)

Freshly collected tissues were fixed in 4% paraformaldehyde, embedded in paraffin, and sectioned for analysis using primary antibodies against Stra8 (rabbit polyclonal, ab49602; Abcam), Ddx4 (rabbit polyclonal ab13840, Abcam; goat polyclonal AF2030, R&D Systems), Sycp3 (rabbit polyclonal NB300-230, Novus Biologicals), Ser^139^-phospho-H2afx (mouse monoclonal 05–636, Millipore) or GFP (chicken polyclonal ab13970, Abcam; rabbit polyclonal ab290, Abcam). For IF, detection was performed using donkey anti-chicken Alexa Fluor 488 (Jackson Immuno), donkey anti-goat Alexa Fluor 647 or donkey anti-rabbit Alexa Fluor 546 (Molecular Probes) as secondary antibody^16^. For IHC, detection was performed using biotin-conjugated anti-rabbit IgG (Santa Cruz Biotechnology) as secondary antibody for horseradish peroxidase-based DAB detection (Sigma-Aldrich). Images were captured using a Nikon E800/BioRad Radiance 2000 confocal microscope or a Nikon ECLIPSE TE2000-S microscope.

### Data analysis

All experiments were independently replicated at least three times, using different mice, tissues collected from different mice, or different populations of cells for each biological replicate. These sample sizes allow for adequate power to detect potential treatment effects while also ensuring that the total number of animals used was minimized. Where possible, assignment of mice to experimental groups was made randomly. Quantitative data from replicate experiments (mean ± s.e.m.) were analyzed by one-way ANOVA followed by Student’s *t*-test (*P* < 0.05), whereas the qualitative images provided are representative of outcomes obtained across the replicate experiments.

## Electronic supplementary material

Supplementary Information
