# Supplementary material for: Genetic studies in mice directly link oocytes produced during adulthood to ovarian function and natural fertility
Source: Sci Rep. 2017 Aug 30;7:10011. doi: 10.1038/s41598-017-10033-6 (PMC5577229; doi:10.1038/s41598-017-10033-6)
Supplement: Supplementary file 1 — Supplementary Information [file 41598_2017_10033_MOESM1_ESM.pdf]

Genetic studies in mice directly link oocytes produced during adulthood to ovarian function and natural fertility

Ning Wang[1,2], Chonthicha Satirapod[1,2], Yasuyo Ohguchi[1,2], Eun-Sil Park[3,4], Dori C. Woods[3] & Jonathan L. Tilly[3]

[1]Vincent Center for Reproductive Biology, Massachusetts General Hospital, and [2]Department of Obstetrics, Gynecology and Reproductive Biology, Harvard Medical School, Boston, Massachusetts 02114, USA. [3]Department of Biology, Laboratory of Aging and Infertility Research, Northeastern University, Boston, Massachusetts 02115, USA

SUPPLEMENTARY INFORMATION

- Supplementary Table S1
- Supplementary Figures S1–S10, with legends

Supplementary Table S1 │ Details for the PCR-based detection of expression of the indicated genes. In addition to sequences of forward and reverse primers used, GenBank accession numbers are provided for all mouse genes, and the predicted size in base pairs of each amplified product is indicated.

Gene	GenBank	Primers	Size
β-actin	NM_007393	Forward, 5'-GATGACGATATCGCTGCGCTG-3'<br>Reverse, 5'-GTACGACCAGAGGCATACAGG-3'	440
Ddx4	NM_010029	Forward, 5'-GGAAACCAGCAGCAAGGGAT-3'<br>Reverse, 5'-TGGAGTCCTCATCCTCTGG-3'	213
Dppa3	NM_139218	Forward, 5'-CCCAATGAAGGACCCTGAAAC-3'<br>Reverse, 5'-AATGGCTCACTGTCCCGTTCA-3'	354
Fabpi (short)	NM_007980	Forward, 5' TGGACAGGACTGGACCTCTGCTTTCCTAGA-3'<br>Reverse, 5'-TAGAGCTTTGCCACATCACAGGTCATTCAG-3'	194
Fabpi (long)	NM_007980	Forward, 5'-CCTCCGGAGAGCAGCGATTAAAAGTGTCAG-3'<br>Reverse, 5'-TAGAGCTTTGCCACATCACAGGTCATTCAG-3'	466
Gfp		Forward, 5'-AAGTTCATCTGCACCACCG-3'<br>Reverse, 5'-TCCTTGAAGAAGATGGTGCG-3'	173
Nobox	NM_130869	Forward, 5'- CCCTTCAGTCACAGTTTCCGT-3'<br>Reverse, 5'- GTCTCTACTCTAGTGCCTTCG-3'	379
Npt		Forward, 5'-TGCTCCTGCCGAGAAAGTATCCATCATGGC-3'<br>Reverse, 5'-CGCCAAGCTCTTCAGCAATATCACGGGTAG-3'	380
Pou5f1	NM_013633	Forward, 5'-CACGAGTGGAAAGCAACTCA-3'<br>Reverse, 5'-AGATGGTGGTCTGGCTGAAC-3'	246
Prdm1	NM_007548	Forward, 5'-GACGGGGGTACTTCTGTTCA-3'<br>Reverse, 5'-GCATCCAGTTGCTTTTCTCC-3'	268
Sohlh1	NM_001001714	Forward, 5'- GATGTCTGTGTACTTCCTCC-3'<br>Reverse, 5'- CTGGCTCACTGAATGACAAC-3'	320
Stra8	NM_009292	Forward, 5'-GCCAGAATGTATTCCGAGAA-3'<br>Reverse, 5'-CTCACTCTTGTCCAGGAAAC-3'	651
Sycp3	NM_011517	Forward, 5'-ACATGGAAAGAAAAGATCTGCTG-3'<br>Reverse,5'-CTGGCTTTGAAAGAAGCTTTG-3'	150
Yfp		Forward, 5'-GCACGACTTCTTCAAGTCCGCCATGCC-3'<br>Reverse, 5'-GCGGATCTTGAAGTTCACCTTGATGCC-3'	280
Zp3	NM_011776	Forward, 5'- CCGAGCTGTGCAATTCCCAGA-3'<br>Reverse, 5'- AACCCTCTGAGCCAAGGGTGA-3'	183

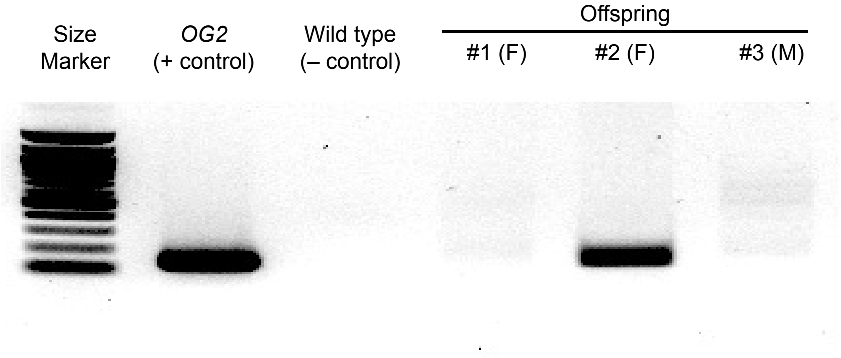

Supplementary Figure S1 │ Intraovarian transplantation of *OG2* transgenic OSCs into wild type female mice leads to birth of *OG2* transgenic offspring. Representative genotype analysis of a litter of three pups (2 female, F; 1 male, M) delivered by one of four wild type female mice that underwent intraovarian transplantation of *OG2* transgenic OSCs followed by natural mating with wild type male mice. The *OG2* transgene was detected in one of the three offspring, indicating its origin from the transplanted cells. Genotype analyses of the parental *OG2* line and of a wild type recipient prior to transplantation are provided as positive and negative controls, respectively. See **Results** section (*Transplanted OSCs generate offspring*) for quantitative data on the total number of litters and offspring analyzed.

a

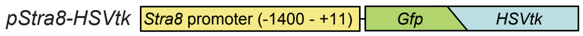

b

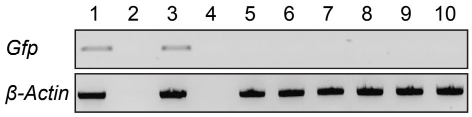

Supplementary Figure S2 │ Generation of *pStra8-HSVtk* transgenic mice. a, Schematics of *Stra8* promoter (*pStra8*)-driven *HSVtk* transgene construct. **b,** Assessment of *Gfp* and *β-actin* mRNA levels in various tissues from adult *pStra8-HSVtk* transgenic knock-in mice: 1, testis; 2, PCR without reverse transcription of testicular RNA sample; 3, ovary; 4, PCR without reverse transcription of ovarian RNA sample; 5, heart; 6, kidney; 7, liver; 8, lung; 9, spleen; 10, brain. Note that the HSVtk construct is a *Gfp*-fusion gene (**a**), and thus *Gfp* is expressed in the *pStra8-HSVtk* transgenic line under the control of the *Stra8* promoter in addition to suicide gene.

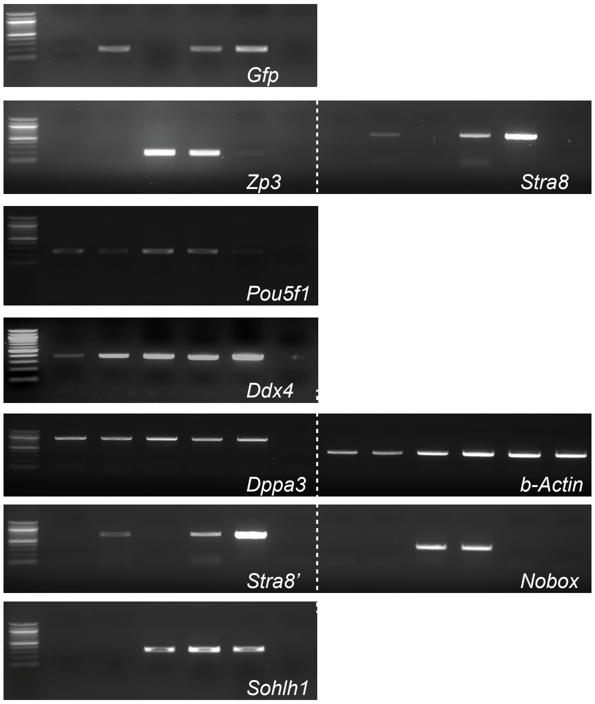

Supplementary Figure S3 │ Gene expression analysis of OSCs, GFP-expressing ovarian cells, oocytes, ovaries, testes, or adult tail-snip fibroblasts isolated from adult *pStra8-Gfp* transgenic mice. Representative uncropped PCR gels presented in Fig. 1d, showing expression of *Stra8*-promoter driven expression of *Gfp*, germ cell markers (*Pou5f1*, *Ddx4*, *Dppa3*), endogenous *Stra8*, oocyte markers (*Nobox*, *Sohlh1*, *Zp3*; *Sohlh1* is also known to be expressed in male germ cells), or β-*actin*.

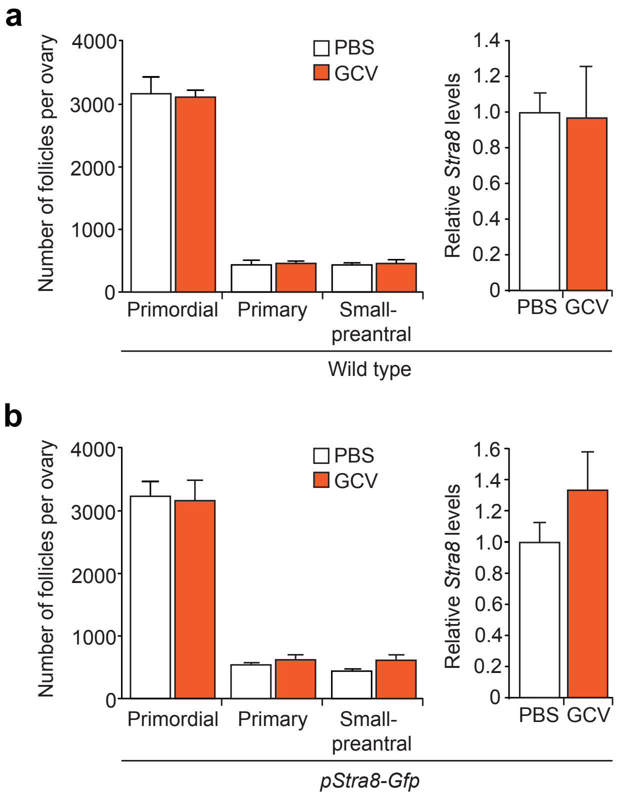

Supplementary Figure S4 │ GCV has no effect on the ovaries in the absence of *Stra8* promoter-driven *HSVtk* expression. a, b, Oocyte-containing follicle numbers and ovarian *Stra8* mRNA levels in wild type (**a**) or *pStra8-Gfp* (**b**) female mice following 21 days of treatment with vehicle (PBS) or GCV (10 mg kg^{-1}) between postpartum days 48–69. See Figure 3 for additional details. Data represent the mean ± s.e.m., *n* = 3–4 mice per group.

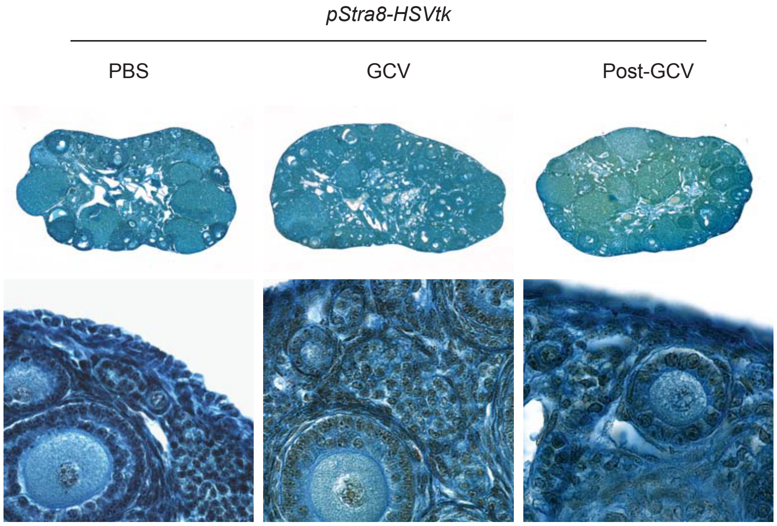

Supplementary Figure S5 │ Gross histological appearance of ovaries of adult *pStra8-HSVtk* females without and with GCV exposure is comparable. Representative histological appearance of ovaries (top, x2; bottom, x40) from adult *pStra8-HSVtk* mice following 21 days of vehicle (PBS) or GCV exposure (10 mg kg^{-1}), or 21 days after cessation of GCV treatment (Post-GCV). Treatments were initiated on postpartum day 48 and stopped on postpartum day 69, with final ovarian collection performed on postpartum day 90. Other than a discernible paucity of primordial-stage follicles (see Fig. 3a for quantitative data), no overt differences in the histological appearance of the ovaries across treatment groups were detected.

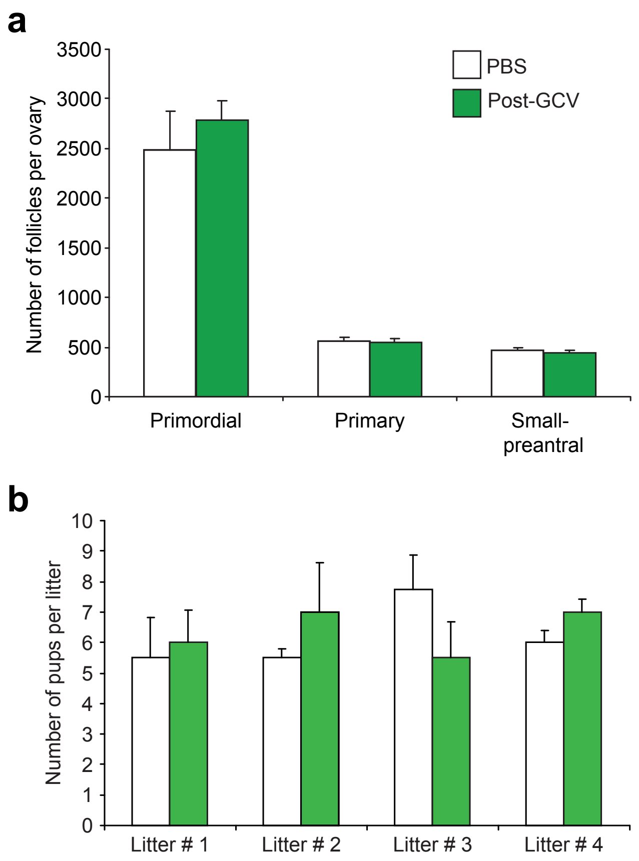

Supplementary Figure S6 │ Long-term stability of oocyte numbers and fertile potential in *pStra8-HSVtk* females following cessation of GCV exposure. a, Comparison of the oocyte-containing follicle numbers in *pStra8-HSVtk* female mice 3 months after completing a 21-day treatment course with vehicle (PBS) or GCV (10 mg kg^{-1}) initiated on postpartum day 48 and stopped on postpartum day 69 (mean ± s.e.m., n = 4–5 mice per group). **b,** Outcomes of natural mating trials (litter size in sequential mating attempts with wild type males over a 6-month period) for *pStra8-HSVtk* female mice after completing a 21-day treatment course with vehicle (PBS) or GCV (10 mg^{-1}) initiated on postpartum day 48 and stopped on postpartum day 69 (mean ± s.e.m., n = 3 mice per group, mated consecutively over 6 months). See panel a for the key to the color-coded bars.

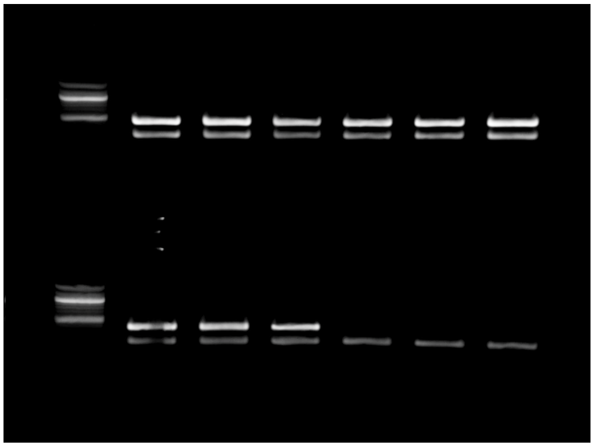

Supplementary Figure S7 │ Genotype analysis of first-generation offspring sired by *pStra8-R26R* males. Representative uncropped PCR gels presented in Fig. 4d, showing that *pStra8-R26R* males induced with Dox (1 mg kg^{-1}) for 28 days before housing with wild type females sire offspring derived from fertilization of wild type eggs by both non-recombined (*Stop* cassette intact) and recombined (*Stop* cassette excised) spermatozoa.

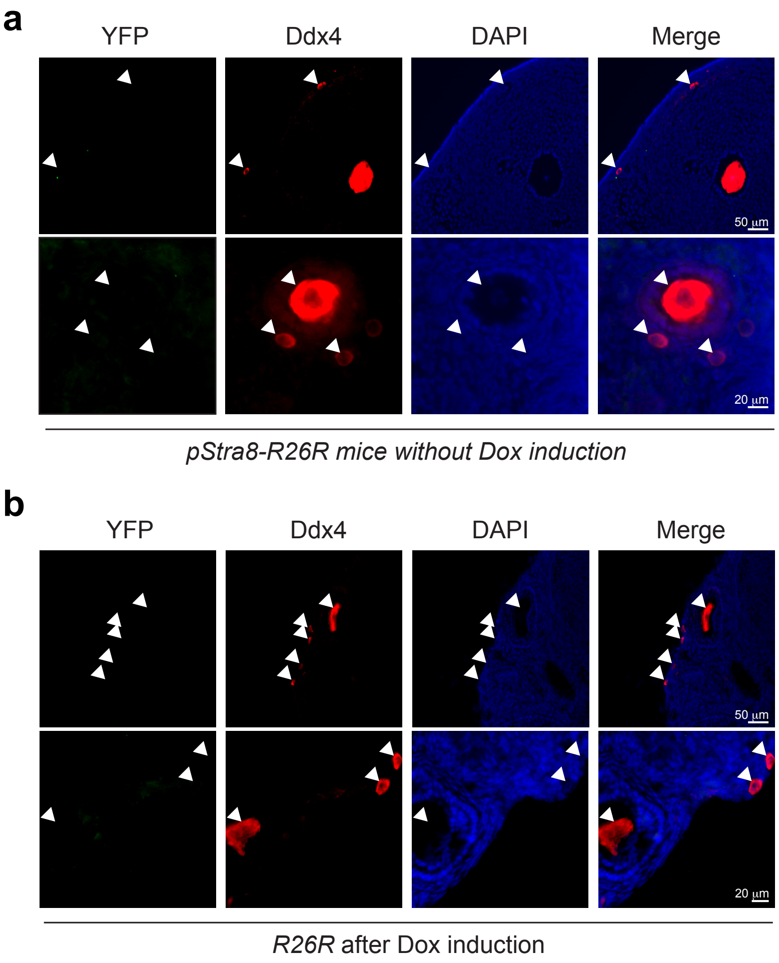

Supplementary Figure S8 │ Expression of YFP is not detectable in *pStra8-R26R* mice without Dox induction or in ovaries of R26R female mice after Dox induction. a, b, Representative expression analysis of YFP (*green*; recombined and activated reporter) and Ddx4 (*red*; germ cell marker) proteins in ovaries of young adult *pStra8-R26R* mice without Dox induction (**a**; injected with vehicle for 21 days) or in ovaries of young adult *R26R* (‘promoterless’ *TRE-Cre*;*Rosa26-Yfp*) mice after 21 days of Dox induction (**b**); white arrowheads demarcate visible primordial and small growing oocytes. These experiments served as negative controls for data on YFP expression presented in Figure 5a.

a

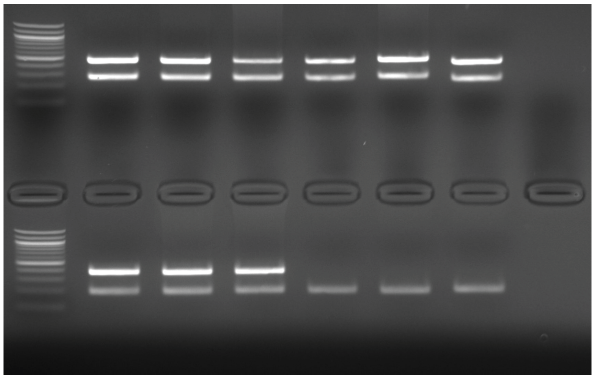

b

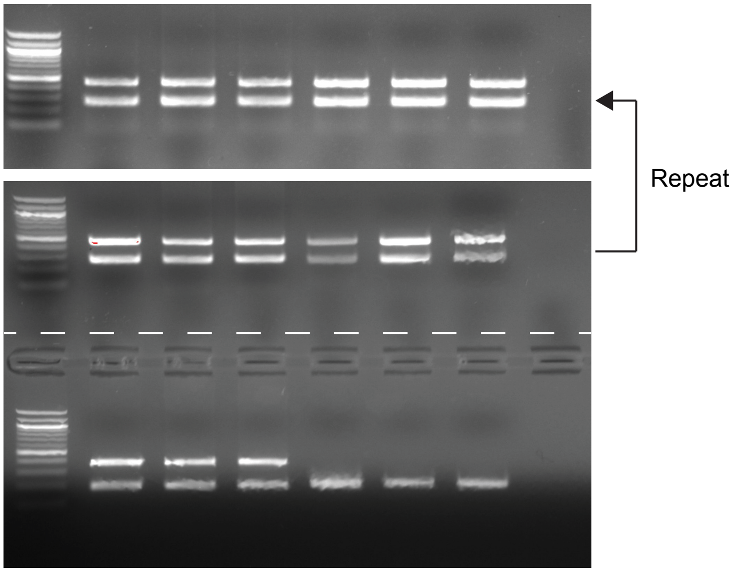

Supplementary Figure S9 │ Genotype analysis of offspring delivered by *pStra8-R26R* females. a, b, Representative uncropped PCR gels presented in Fig. 5b (**a**) and Fig. 5d (**b**), showing that *pStra8-R26R* females induced with Dox (10 mg kg^{-1}) for 21 days before housing with wild type males deliver offspring derived from fertilization of both non-recombined (*Stop* cassette intact) and recombined (*Stop* cassette excised) eggs (**a**; F_1 generation), and that the recombined F_1 offspring successfully transmit the recombined locus to F_2 offspring in natural mating trials (**b**).

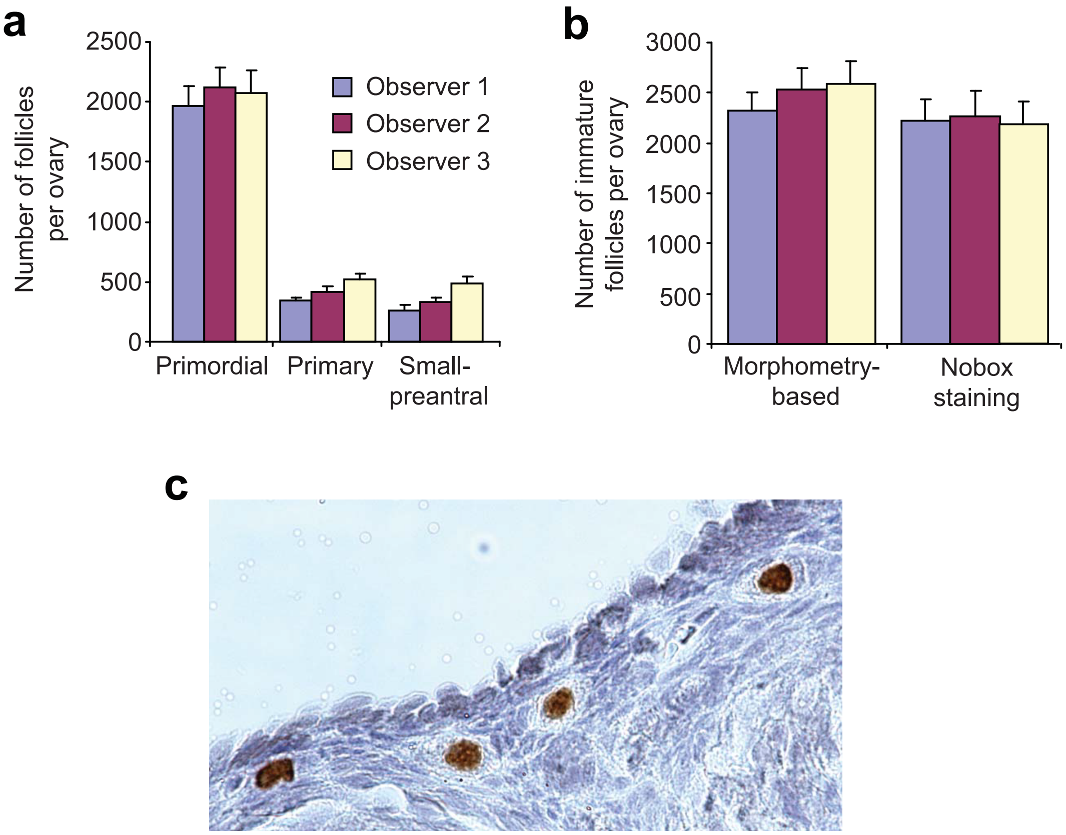

Supplementary Figure S10 │ Verification of the accuracy and reproducibility of the oocyte counting protocol. a, Adult mouse ovaries were fixed, paraffin-embedded, serially-sectioned, aligned in order on glass slides, vital dye-stained, and assessed by three observers in a blinded manner by histomorphometry to estimate the number of immature oocyte-containing follicles at the resting (primordial) and early growing (primary, small-preantral) stages of development. Each observer was randomly assigned a different start section from one of the first five sections, and every fifth sequential section was then analyzed by light microscopy. No differences in counts across observers were detected (mean ± s.e.m., *n* = 3 mice). **b,** Ovaries contralateral to those used for the follicle counts in panel **a** were fixed, paraffin embedded, serially sectioned, and processed for immunohistochemical detection of Nobox, which is specifically expressed in immature oocytes. Three observers then independently assessed the slides in a blinded manner to estimate the number of primordial and primary follicles containing Nobox-positive oocytes. Each observer was randomly assigned a different start section from one of the first five sections, and every fifth sequential section was then analyzed by light microscopy. No differences in Nobox-positive oocyte counts across observers (mean ± s.e.m., *n* = 3 mice), or across the method of oocyte detection (histological versus immunohistochemical), were found. See panel a for the key to the color-coded bars. **c,** Example of the specificity of Nobox immunostaining (*brown*, against a *blue* hematoxylin counterstain).
